# Do Publicly Available Risk Calculators Apply to Adult Spinal Deformity Surgery?

**DOI:** 10.3390/jcm14248618

**Published:** 2025-12-05

**Authors:** Anthony Pajak, Justin T. Samuel, Tejas Subramanian, Robert K. Merrill, Izzet Akosman, John C. Clohisy, Jerry Du, Bo Zhang, Jonathan Elysee, Pratyush Shahi, David N. Kim, Yusef Jordan, Rachel L. Knopp, Francis C. Lovecchio, Han Jo Kim

**Affiliations:** 1 Department of Spine Surgery, Hospital for Special Surgery, 535 E. 71st Street, New York, NY 10021, USAiakosman@houstonmethodist.org (I.A.); clohisyj@gmail.com (J.C.C.); elysee.jonathan@gmail.com (J.E.);; 2 UMass Chan Medical School, 55 N Lake Avenue, Worcester, MA 01655, USA; 3 Weill Cornell Medical Center, 525 East 68th Street, New York, NY 10065, USA

**Keywords:** spinal deformities, spinal fusion, risk assessment, prognostic models, postoperative complications, outcome assessment, national surgical quality improvement program

## Abstract

**Background/Objectives**: The American College of Surgeons National Surgical Quality Improvement Program (ACS-NSQIP) and SpineSage^TM^ risk calculators are automated online tools to predict short-term complications for surgical procedures. The objective of this study was to assess the validity of ACS-NSQIP and SpineSage^TM^ risk calculators to predict short-term complications after adult spinal deformity (ASD) surgery. **Methods**: We included ASD patients who had surgery between 2017 and 2020 (≥5 levels, single-stage, posterior-only). Patient factors were entered into the risk calculators to generate probabilities for 30-day outcomes. Calibration and discrimination were assessed using Brier scores and C-statistics, respectively. **Results**: A total of 198 patients were included (67 male, 131 female) who underwent posterior spinal fusion for ASD surgery. The ACS-NSQIP risk calculator had strong calibration for all complications (Brier score < 0.09) except non-home discharge (Brier score 0.2). Discrimination was poor for all complications except surgical site infection (C-statistic 0.86), venous thromboembolism (C-statistic 0.84), and readmission (C-statistic 0.7). The SpineSage^TM^ risk calculator had strong calibration for all complications (Brier score < 0.09) aside from the “any complications” subset (Brier score 0.36). The discrimination capacity was poor for all complications (C-statistic < 0.7). **Conclusions**: The ACS-NSQIP calculator had strong calibration and poor discrimination for most complications. The SpineSage^TM^ calculator had strong calibration for most complications and a poor discrimination capacity for all complications. NSQIP calculation deficits may be due to the reliance on a single CPT code to calculate risk. The deficient discriminatory capacity of the SpineSage^TM^ calculator may be due to the inclusion of common perioperative occurrences as complications.

## 1. Introduction

Shared decision-making has become a cornerstone of patient care throughout all specialties in medicine, consistently demonstrating improved patient centered outcomes and satisfaction [1,2]. To effectively engage in shared decision-making, the surgeon and patient must adequately understand the risks associated with undergoing a procedure or treatment. The risk of complications following adult spinal deformity (ASD) surgery can be as high as 67.4% [3]. Patient age, comorbidities, and surgical invasiveness have been reported to increase these risks [4,5,6,7]. In addition to guiding presurgical counseling, surgical risk calculators may also help foster a cost-conscious healthcare landscape through the stratification of high-risk ASD patients [8]. Various automated, publicly available online risk calculators aim to use patient demographic and surgical characteristics to estimate the risk of developing postoperative complications. However, a 2024 systematic review highlighted the lack of externally validated risk stratification models specific for spine surgery [9]. Two such tools are the American College of Surgeons National Surgical Quality Improvement Program (ACS-NSQIP) and SpineSage^TM^ risk calculators.

The ACS-NSQIP risk calculator has been deemed an adequate predictive tool for a subset of complications following anterior lumbar interbody fusion (ALIF) and primary cervical fusion procedures [10,11]. However, the calculator was deficient in predicting specific complications in a more granular manner. Few investigations have validated this tool for ASD corrective surgery [12]. The SpineSage^TM^ risk calculator is specific to spine surgery and has been shown to more accurately predict the risk of serious medical complications in comparison to the ACS-NSQIP risk calculator following single-stage spine surgery [13]. However, minimal literature exists evaluating its ability to predict complications following surgery for ASD [14]. Accordingly, the primary outcome of this study is to validate the ability of the ACS-NSQIP and SpineSage^TM^ risk calculators to predict short term complications after ASD surgery by a single surgeon.

## 2. Materials and Methods

### 2.1. Patient Population and Data Collection

This was a single center retrospective cohort study approved by the Institutional Review Board (IRB). Patients were retrospectively queried from a prospectively maintained single surgeon registry. The patient inclusion criteria were as follows:

Inclusion criteria:

- Adult patients (age > 18 years) with spinal deformity.
- Patients undergoing primary, posterior-only, single-stage fusion procedures.
- Fusion involving more than five levels, with or without extension to the pelvis.
- Surgery performed between 1 January 2017 and 31 December 2020.

Exclusion criteria:

- Age < 18 years.
- Neuromuscular, infectious, or post-traumatic deformities.
- Incomplete preoperative demographic and comorbidity data.
- Insufficient 30-day postoperative follow-up to assess complications of interest.
- Concurrent surgeries outside the spine.

The following subsections demonstrate the patient data that was collected in order to perform risk calculations utilizing the 2022 versions of the ACS-NSQIP and SpineSage^TM^ calculators. The data was collected and managed using REDCap (Research Electronic Data Capture) [15] hosted at Weill Cornell Medicine Clinical and Translational Science Center supported by the National Center for Advancing Translational Science of the National Institute of Health under award number UL1 TR002384:

1. Demographic details: age, gender, body mass index (BMI), American Society of Anesthesiologists (ASA) class, Charlson Comorbidity Index (CCI), and any individual comorbidities (hypertension, diabetes, kidney disease, heart disease, liver disease, vascular disease, autoimmune disease, rheumatological disease, dyslipidemia, cancer, peptic ulcer disease, osteoarthritis/osteoporosis, peripheral neuropathy, bleeding/hypercoagulable disorder, anxiety/depression).
2. Operative details: number of levels fused, 3-column osteotomy, operative time, estimated blood loss (EBL).
3. Peri and postoperative details: length of stay, any complications (pneumonia, cardiac, surgical site infection, urinary tract infection, sepsis, venous thromboembolism, renal failure, blood loss requiring transfusion, dural tear), readmission, reoperation, non-home discharge.

### 2.2. Risk Calculators Data Collection

**Figure 1:**
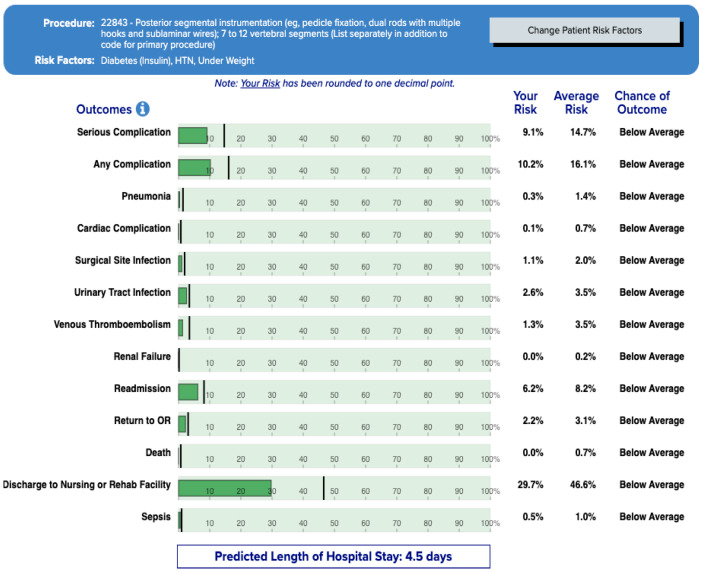
Sample outputs of the ACS-NSQIP surgical risk calculator report screen. Estimated risks for 11 complication categories, length of stay, and discharge to a nursing or rehab facility are displayed following the input of the CPT code for the planned procedure and patient specific variables.

**Figure 2:**
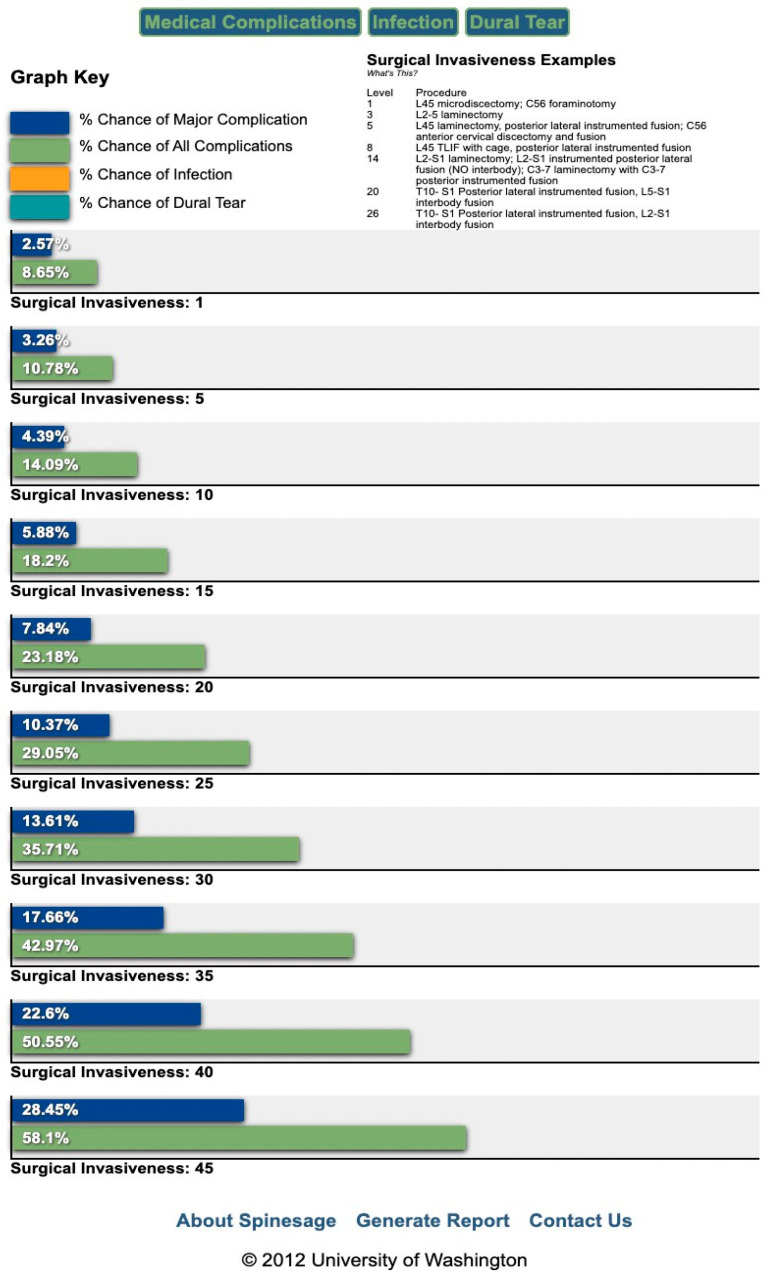
Sample outputs of the SpineSage^TM^ surgical risk calculator report screen. Estimated risks for major complication, all complications, infection, and dural tear are displayed based on surgical invasiveness scores and patient specific variables.

Patient characteristics and demographics were entered into each risk calculator. CPT codes for the ACS-NSQIP calculator were selected to best match the type and attributes of the surgical procedures performed. The CPT codes included 22843 (posterior instrumentation 7–12 levels), 22844 (posterior instrumentation 13+ levels), 22206 (thoracic three column osteotomy), and 22207 (lumbar three column osteotomy), all used when appropriate. Sample output report sheets of the ACS-NSQIP and SpineSage^TM^ risk calculators are displayed in Figure 1 and Figure 2, respectively. Risk profiles were recorded for each patient. The SpineSage^TM^ risk calculator also attempts to capture surgical complexity by categorizing the risk profile based on a surgical invasiveness score developed by Mirza et al. [16]. We calculated this invasiveness score and used the appropriate risk profile.

### 2.3. Statistical Analysis

All statistical analyses were performed using Posit Team (2023). RStudio: Integrated Development Environment for R. Posit Software version 2023.03.0, PBC, Boston, MA. URL http://www.posit.co/ (accessed on 14 July 2025). The risk calculators were evaluated for calibration and discrimination ability using the Brier score and C-statistic, respectively. Briefly, the Brier score is used to determine the difference between the predicted outcome and the actual outcome for each individual observation, with a perfect score being 0 and the worst possible score being 1 [17]. The c-statistic measures discrimination and is also known as the area under the curve (AUC) of the receiver operating characteristic curve (ROC). Briefly, discrimination is the chance that a random participant who did in fact develop the outcome (complication) had a higher risk probability than a participant who did not develop the outcome of interest. There are various interpretations of the output, but generally a value of greater than or equal to 0.7 is considered acceptable [18].

We also divided patients into cohorts of those who experienced a complication as defined by each risk calculator and those who did not and compared various demographics that were input into the risk calculators. The definition of a complication for the NSQIP risk calculator can be found on the website (https://riskcalculator.facs.org/RiskCalculator/ (accessed on 14 July 2025)). The definitions of medical complications of the SpineSage^TM^ risk calculator are in accordance with those reported by Lee et al. [14]. A summary of the ACS-NSQIP and SpineSage^TM^ specific complication and outcome of interest definitions is listed in Table 1.

All continuous variables were assessed for normality with a Shapiro–Wilk’s normality test. Normally distributed continuous variables were compared with a two-tailed independent sample *t*-test, while non-normally distributed variables were compared with a Wilcoxon rank sum test. Categorical variables were compared between groups with a chi-square test or a Fisher’s exact test when applicable. Statistical significance was taken at *p*-value < 0.05.

## 3. Results

### 3.1. Patient Cohort Demographics and Complication Profiles

A total of 198 patients met the inclusion criteria, comprising 67 (34%) males and 131 (66%) females (Table 2). The mean age was 61.0 ± 15.68 years, and the mean BMI was 27 ± 6.07. Most patients were classified as ASA class II (69%) or ASA class III (30%) with a mean CCI of 2.3 ± 1.61 (Table 2). Patients had an average of 9 ± 3.26 levels fused, with 26% undergoing a three-column osteotomy. The mean operative time was 260 ± 81.88 min, and the mean estimated blood loss was 1.1 ± 0.80 L.

Patient demographics and the ACS-NSQIP-defined complication profiles are summarized in Table 2. Although the ACS-NSQIP and SpineSage^TM^ calculators apply different thresholds for defining complications, the demographic patterns of patients with and without complications were broadly comparable. Across both definitions, operative time, estimated blood loss (EBL), number of levels fused, and length of stay were all higher for those that had complications. The ASA class was significantly higher for those that had complications under the ACS-NSQIP definition, while age, hypertension, and lung disease were significantly higher in those who had complications under the SpineSage^TM^ definition. Patient demographics and the SpineSage^TM^ defined complication profiles are summarized in Table 3.

### 3.2. ACS-NSQIP Calculator

Brier scores for overall, overall serious complications, pneumonia, cardiac complications, surgical site infection (SSI), urinary tract infection (UTI), venous thromboembolism (VTE), renal failure, readmission, re-operation, and death were calculated. All demonstrated good calibration in the prediction of event risk utilizing the ACS-NSQIP calculator. Non-home discharge was the single variable for which the ACS-NSQIP Brier score demonstrated less calibration (Brier score 0.2). Discriminatory performance was poor (<0.7) for all outcomes except SSI, VTE, and readmission (Table 4). The median predicted length of stay (5 days ± 1.5) was statistically shorter than the observed length of stay (5.4 ± 3) (*p* = 0.03).

### 3.3. SpineSage^TM^ Calculator

The SpineSage^TM^ demonstrated proficient calibration for predicting serious complications, infection (Brier score 0.02 for both), and dural tear (Brier score 0.1). However, the discriminatory performance was poor for all complications (C-statistic < 0.7). Moreover, the predicted event rates differed substantially from observed incidences, with the exception of dural tear (Table 5).

## 4. Discussion

Understanding the risk patients face when undergoing certain procedures and treatments is integral to the shared decision-making process. Accurate predictive models can provide both surgeons and patients with a clearer understanding of potential complications. In this retrospective review, we evaluated the performance of the ASC-NSQIP and SpineSage^TM^ risk calculators to predict short term complications after adult spinal deformity surgery. The ACS-NSQIP risk calculator had good accuracy, also termed calibration, as determined by the Brier score for all complications (Brier < 0.1) except for non-home discharge (Brier = 0.2). In terms of discrimination, also known as the area under the ROC, the calculator performed well (C-statistic ≥ 0.7) only for predicting surgical site infection, venous thromboembolism, and readmission. The SpineSage^TM^ risk calculator demonstrated strong calibration for major complications, SSI, and dural tear (Brier ≤ 0.1), but the discrimination capacity was poor for all predicted outcomes (C-statistic < 0.7).

With regard to adult spinal deformity specifically, only a few studies have investigated the performance of the NSQIP risk calculator [12,19,20]. A study by Pierce et al. assessed the calculator using the Brier score and found that the calculator was accurate (Brier < 0.25) for predicting all the evaluated complications [12]. This supports our results based on Brier score alone. Interestingly, the least accurate outcome predicted in their cohort was non-home discharge (Brier = 0.19), similarly to our cohort (Brier = 0.2). A Brier score of 0.25 is equal to randomness, and so while these results technically suggest better than random calibration, they are not good scores. This is further supported by both Pierce et al. and our results showing that the predicted incidence of non-home discharge is vastly different from the actual incidence of non-home discharge [12]. A 2024 cohort study of ASD patients demonstrated similar findings, with poor prediction of non-home discharge, SSI, reoperation, and overall complications [13]. The predicted and actual incidence in both studies for several of the complications are within one percentage point. This suggests the calculator may be beneficial as a benchmarking tool in counseling patients on the incidence of complications in a population with similar demographics and surgical characteristics as their own. The SpineSage^TM^ has not been extensively studied in adult spinal deformity surgery but has been shown to have good discrimination and superior predictive capacity for major complications in other single stage spinal surgery applications [19,20]. Consistent with our findings, Jadresic et al. studied the SpineSage^TM^ tool on a heterogeneous population and found that the discrimination was poor for detecting major complication (Brier = 0.19), SSI (Brier = 0.08), and dural tear (Brier = 0.14), only being proficient for any complication (Brier = 0.19) [21].

The current literature assessing the validity of the NSQIP and SpineSage^TM^ risk calculators is not ubiquitous. McCarthy et al. evaluated the NSQIP risk calculator for elective cervical and lumbar fusions [11]. They only evaluated the calculator using the area under the ROC (c-statistic) and found in the lumbar fusion cohort it only had good discrimination for predicting pneumonia. It is important to note, though, they set a more stringent cutoff of >0.8. If their cutoff for acceptable performance was set at the same as ours, ≥0.7, the calculator had good discrimination for acute renal failure as well. Both pneumonia and acute renal failure only occurred at a <1% incidence, and therefore the authors appropriately caution the results may not be fully reflective of the model’s discriminatory capacity. Another study by Neassig et al. highlights important limitations of the NSQIP risk calculator when predicting complications for adult spinal deformity patients, namely that it lacks granularity of each patient and procedure [19]. When patients were stratified by frailty, there was a 71% incidence of actual complications but only a 17% predicted incidence, with a Brier score of 0.37. These findings clearly demonstrate the calculator is inaccurate and should be used judiciously in certain patient populations. They saw similar results when stratifying patients by high T1 pelvic angle (TPA) or pelvic incidence–lumbar lordosis (PI–LL), for every complication assessed. These findings demonstrate that the complexity of a surgical procedure simply cannot be captured by the NSQIP risk calculator. Similarly, we found that the NSQIP risk calculator demonstrated acceptable discrimination only for SSI, VTE, and readmission. All of which are outcomes largely influenced by systemic factors rather than the detailed clinical variables that the calculator fails to capture. With respect to SpineSage^TM^, conflicting findings have been reported as well. Kasparek et al. evaluated the risk calculator on a heterogeneous patient population of cervical, thoracic, and lumbar patients indicated for degenerative, traumatic, infectious, and neoplastic conditions. They reported a predicted (14.7%) and actual (16.1%) incidence of overall complications and a strong discrimination with a c-statistic of 0.71 for all and 0.85 for major complications [22].

Accurate predictive models must demonstrate both excellent calibration and discrimination; adequacy in one or the other domain is insufficient. Few studies have investigated the NSQIP and SpineSage^TM^ tools in elective spine surgery. Future studies should assess the validity of the NSQIP and SpineSage^TM^ risk calculators exclusively in adult spinal deformity patient populations in order to draw fair comparisons. To remediate the reported deficits in discriminatory capacity of the NSQIP tool, future developments must incorporate specific surgical parameters to adequately account for the invasiveness of ASD surgery, thereby improving predictive modeling for patients. The SpineSage^TM^ calculator attempts to overcome the previously mentioned flaws by instituting a spine specific surgical invasiveness score developed by Mirza et al. [16]. This score incorporates the number of levels decompressed and instrumented from both a posterior and anterior approach and is input into the calculator to generate a more accurate risk profile. SpineSage^TM^ has been shown to have good discrimination for more heterogenous cohorts of spine procedures [20,22]. We feel the inaccuracies in SpineSage^TM^ may come from two specific drawbacks. The first is that EBL > 3L and transfusion occurrence are considered complications by the calculator. For corrective spinal deformity procedures, these are not uncommon occurrences. Our cohort had a 62% incidence of transfusions, all of which were technically considered a complication according to the calculator. Additionally, the surgical invasiveness score will be very high for deformity correction procedures, which may overestimate the prediction of complications by the calculator. While deformity procedures are certainly invasive, the magnitude of increase in the invasiveness score simply by instrumenting additional levels may not correlate with an actual increased risk of developing complications. These two points highlight that the calculator may not be suited for adult deformity patients. Future developments should tailor reasonable definitions of complications and invasiveness in accordance with spinal deformity correction.

A major limitation of our study is that it was a single surgeon cohort. Including more surgeons would make the calculator more generalizable and less specific to one person. The study was retrospective in nature, which predisposes it to inherent bias and possible inaccuracy in data collection. Lastly, we did not have an occurrence of certain medical complications, which limited our ability to evaluate the NSQIP calculator with respect to predicting those specific complications.

## 5. Conclusions

Our results demonstrate that the ACS-NSQIP calculator had strong calibration and poor discrimination for most complications. The SpineSage^TM^ calculator had strong calibration for most complications and a poor discrimination capacity for all complications. For the ACS-NSQIP risk calculator, this likely relates to the use of a single CPT code for procedure input, which underestimates the complexity of adult spinal deformity. While the SpineSage^TM^ risk calculator attempts to capture the complexity of the surgical procedure, it may be set back by including a variety of common occurrences after such procedures in its definition of a complication. Thus, these calculators may be more appropriate as benchmarking tools rather than utilized for individual patient risk counseling.

## Figures and Tables

**Table 1:** Complication and outcome of interest definitions as defined by the ACS-NSQIP and SpineSage^TM^ risk calculators.

Complication	ACS-NSQIP Definition	SpineSage^TM^ Definition
Any Complication	Superficial incisional SSI, deep incisional SSI, organ space SSI, wound disruption, pneumonia, unplanned intubation, PE, ventilator > 48 h, progressive renal insufficiency, acute renal failure, UTI, stroke, cardiac arrest, myocardial infarction, DVT, return to the operating room, systemic sepsis	GI bleeding, ileus, obstruction, pancreatitis, perforation, peritonitis, other GI occurrence, CVA/TIA, cerebral perfusion, delirium, diabetes insipidus, electrolyte change, meningitis, SAH/intracerebral hemorrhage, seizure, alcohol withdrawal, narcotic withdrawal, coagulopathy, EBL > 3 L, transfusion occurrence, Foley catheter trauma, renal insufficiency, urinary retention, other neurologic event
Serious/Major Complications	Cardiac arrest, myocardial infarction, pneumonia, progressive renal insufficiency, acute renal failure, PE, DVT, return to the operating room, deep incisional SSI, organ space SSI, systemic sepsis, unplanned intubation, UTI, wound disruption	Cardiac arrest, myocardial infarction, myocardial ischemia, acute respiratory distress syndrome, postoperative hypoxia, pulmonary embolus, respiratory arrest, GI perforation, CVA/TIA, meningitis, SAH/intracerebral hemorrhage
Pneumonia	Pneumonia	Infection of the lung parenchyma confirmed by fever, sputum or bronchial cultures, CXR, and requiring treatment
Cardiac Complications	Cardiac arrest or MI	Air embolism, cardiac arrest, arrhythmia, CHF, hypertension, hypotension, myocardial infarction, inappropriate or inadequate fluid therapy, myocardial ischemia, thermoregulation, other cardiac occurrence
Surgical Site Infection	Surgical site infection	-
Urinary Tract Infection	Urinary tract infection	The presence of large amounts of bacteria (>100,000 organisms/mL) in the upper or lower urinary tract associated with symptoms or requiring treatment
Venous Thromboembolism	Venous thromboembolism	The presence of thrombosis of the iliac, femoral, popliteal, or other veins confirmed by imaging studies (duplex scan, CT, or MR) with or without swelling, warmth, erythema, or tenderness
Renal Failure/Insufficiency	Progressive renal insufficiency or acute renal failure	Operational definition: Failure of the kidneys characterized by rapid decline in glomerular filtration rate (hours to days), retention of nitrogenous waste products, and perturbation of extracellular fluid volume and electrolyte and acid–base homeostasis; criteria: Serum Cr > 2 above baseline
Readmission	Readmission	-
Reoperation	Return to OR	-
Non-Home Discharge	Discharge to nursing or rehab facility	-
Death	Death	Death
Length of Stay (median days)	Predicted length of hospital stay	-
Dural Tear	-	Dural tear
Infection	-	Surgical site infection

**Table 2:** Patient demographics and complication profiles as defined by the ACS-NSQIP risk calculator.

ACS-NSQIP Defined Complications				
Variable	Total (198)	No Complication (*n* = 180)	Any Complication (*n* = 18)	*p*-Value
Sex (*n*, %)				0.878
Sex: Male	67 (33.8%)	62 (34.4%)	5 (27.8%)	
Sex: Female	131 (66.2%)	118 (65.6%)	13 (72.2%)	
Age (mean, sd)	60.68 (15.68)	60.98 (15.72)	57.67 (15.40)	0.251
BMI (mean, sd)	27.11 (6.07)	26.93 (6.10)	28.97 (5.51)	0.096
Current/Former Smoker (*n*, %)	4 (2.0%)	4 (2.2%)	0 (0.0%)	0.645
Medical Comorbidities (*n*, %)				
Hypertension	38 (19.2%)	35 (19.4%)	3 (16.7%)	0.717
Diabetes	16 (8.1%)	15 (8.3%)	1 (5.6%)	0.825
Kidney Disease	4 (2.0%)	3 (1.7%)	1 (5.6%)	0.382
Lung Disease	6 (3.0%)	5 (2.8%)	1 (5.6%)	0.346
Congestive Heart Failure	2 (1.0%)	2 (1.1%)	0 (0.0%)	0.732
Myocardial Infarction	3 (1.6%)	3 (1.7%)	0 (0.0%)	0.855
Coronary Artery Disease	8 (4.3%)	6 (3.3%)	2 (11.1%)	0.275
Cerebrovascular Disease	4 (2.0%)	4 (2.2%)	0 (0.0%)	0.813
Peripheral Vascular Disease	2 (1.0%)	2 (1.1%)	0 (0.0%)	0.9
Autoimmune Disease	8 (4.3%)	8 (4.4%)	0 (0.0%)	0.658
Dyslipidemia	48 (25.5%)	43 (23.9%)	5 (27.8%)	0.924
Cancer	18 (9.6%)	17 (9.4%)	1 (5.6%)	0.859
Liver Disease	2 (1.0%)	2 (1.1%)	0 (0.0%)	0.9
Peptic Ulcer Disease	3 (1.6%)	3 (1.7%)	0 (0.0%)	0.855
Gastroesophageal Reflux Disease	47 (23.7%)	43 (23.9%)	4 (22.2%)	0.984
Osteoarthritis	20 (10.6%)	18 (10.0%)	2 (11.1%)	0.982
Osteoporosis	30 (16%)	26 (14.4%)	4 (22.2%)	0.668
Rheumatologic Disease	7 (3.7%)	7 (3.9%)	0 (0.0%)	0.694
Anxiety	25 (13.3%)	25 (13.9%)	0 (0.0%)	0.239
Depression	14 (7.4%)	14 (7.8%)	0 (0.0%)	0.471
Peripheral Neuropathy	3 (1.6%)	3 (1.7%)	0 (0.0%)	0.855
Hypercoagulability Disorder	4 (2.0%)	3 (1.7%)	1 (5.6%)	0.53
Bleeding Disorder	3 (1.6%)	3 (1.7%)	0 (0.0%)	0.855
American Society of Anesthesiologists Class (*n*, %)				**0.027**
ASA I	3 (1.6%)	2 (1.1%)	1 (5.6%)	
ASA II	136 (68.7%)	128 (71.1%)	8 (44.4%)	
ASA III	59 (29.8%)	50 (27.8%)	9 (50.0%)	
Charlson Comorbidity Index (mean, standard deviation)	2.31 (1.61)	2.36 (1.63)	1.89 (1.41)	0.318
Operative and Perioperative Data				
Three-Column Osteotomy (*n*, %)	52 (26.3%)	47 (26.1%)	5 (27.8%)	1
Operative Time (mean hours, standard deviation)	259.95 (81.88)	255.41 (79.33)	305.35 (95.07)	**0.028**
Estimated Blood Loss (mean, standard deviation)	1070.36 (801.3)	1033.45 (793.46)	1433.33 (809.68)	**0.029**
Number of Levels Fused (mean, standard deviation)	8.96 (3.26)	8.82 (3.04)	10.39 (4.82)	0.405
Length of Stay (mean, standard deviation)	6.20 (3.27)	6.05 (3.32)	7.70 (2.29)	**0.001**

Note: Bold indicates that *p* < 0.05, the data is statistically significant.

**Table 3:** Patient demographics and complication profiles as defined by the SpineSage^TM^ risk calculator.

SpineSage^TM^ Defined Complications				
Variable	Total (198)	No Complication (*n* = 63)	Any Complication (*n* = 135)	*p*-Value
Sex (*n*, %)				
Sex: Male	67 (33.8%)	26 (41.3%)	41 (30.4%)	0.284
Sex: Female	131 (66.2%)	37 (58.7%)	94 (69.6%)	
Age (mean, standard deviation)	60.68 (15.68)	56.43 (18.16)	62.66 (14.01)	**0.024**
Body Mass Index (mean, standard deviation)	27.11 (6.07)	27.65 (6.49)	26.85 (5.87)	0.532
Current or Former Smoker (*n*, %)	4 (2%)	1 (1.6%)	3 (2.2%)	0.37
Medical Comorbidities (*n*, %)				
Hypertension	38 (19.2%)	5 (7.9%)	33 (24.4%)	**0.021**
Diabetes	16 (8.1%)	3 (4.8%)	13 (9.6%)	0.303
Kidney Disease	4 (2%)	2 (3.2%)	2 (1.5%)	0.223
Lung Disease	6 (3%)	3 (4.8%)	3 (2.2%)	**0.035**
Congestive Heart Failure	2 (1%)	0 (0.0%)	2 (1.5%)	0.256
Myocardial Infarction	3 (1.6%)	1 (1.6%)	2 (1.5%)	1
Coronary Artery Disease	8 (4.3%)	2 (3.2%)	6 (4.4%)	0.967
Cerebrovascular Disease	4 (2%)	0 (0.0%)	4 (3%)	0.4
Peripheral Vascular Disease	2 (1%)	1 (1.6%)	1 (0.7%)	1
Autoimmune Disease	8 (4.3%)	4 (6.3%)	4 (3%)	0.463
Dyslipidemia	48 (25.5%)	18 (28.6%)	30 (22.2%)	0.434
Cancer	18 (9.6%)	5 (7.9%)	13 (9.6%)	0.896
Liver Disease	2 (1%)	1 (1.6%)	1 (0.7%)	1
Peptic Ulcer Disease	3 (1.6%)	1 (1.6%)	2 (1.5%)	1
Gastroesophageal Reflux Disease	47 (23.7%)	15 (23.8%)	32 (23.7%)	1
Osteoarthritis	20 (10.6%)	8 (12.7%)	12 (8.9%)	0.571
Osteoporosis	30 (16%)	8 (12.7%)	22 (16.3%)	0.646
Rheumatological Disease	7 (3.7%)	2 (3.2%)	5 (3.7%)	1
Anxiety	25 (13.3%)	8 (12.7%)	17 (12.6%)	1
Depression	14 (7.4%)	6 (9.5%)	8 (5.9%)	0.539
Peripheral Neuropathy	3 (1.6%)	1 (1.6%)	2 (1.5%)	1
Hypercoagulability Disorder	4 (2%)	0 (0.0%)	4 (3%)	0.4
Bleeding Disorder	3 (1.6%)	0 (0.0%)	3 (2.2%)	0.568
American Society of Anesthesiologists Class (*n*, %)				0.099
ASA Class I	3 (1.6%)	1 (1.6%)	2 (1.5%)	
ASA Class II	136 (68.7%)	49 (77.8%)	87 (64.4%)	
ASA Class III	59 (29.9%)	13 (20.6%)	46 (34.1%)	
Charlson Comorbidity Index (mean, standard deviation)	2.31 (1.61)	2.02 (1.82)	2.45 (1.49)	**0.026**
Operative and Perioperative Data				
Three-Column Osteotomy	52 (26.3%)	12 (19.0%)	40 (29.6%)	0.161
Operative Time (hours, standard deviation)	259.95 (81.88)	219.49 (73.91)	278.83 (78.74)	**<0.001**
Estimated Blood Loss (mean, standard deviation)	1070.36 (801.30)	876.29 (619.66)	1160.83 (860.40)	**0.026**
Number of Levels Fused (mean, standard deviation)	8.96 (3.26)	7.81 (2.31)	9.50 (3.50)	**0.004**
Length of Stay (mean, standard deviation)	6.20 (3.27)	5.07 (2.27)	6.73 (3.53)	**0.001**

Note: Bold indicates that *p* < 0.05, the data is statistically significant.

**Table 4:** Complication predictions produced by the ACS-NSQIP risk calculator.

Variable	Predicted Probability	Actual Incidence	Brier Score	C-Statistic
Any Complication	15.2	9.1	0.09	0.57
Serious Complications	13.2	8.6	0.08	0.57
Pneumonia	1.4	0.5	0.005	0.51
Cardiac	0.8	0	0.0001	-
Surgical Site Infection	2.2	2	0.02	0.86
Urinary Tract Infection	3.2	3.5	0.03	0.56
Venous Thromboembolism	2.6	0.5	0.006	0.84
Renal Failure	0.23	0	0.00002	-
Readmission	6.1	3	0.03	0.7
Reoperation	3.7	2.5	0.02	0.57
Non-Home Discharge	41.6	10.2	0.2	0.58
Death	0.4	0	0.00005	-
Length of Stay (median days)	5	5.4	-	-

Note: Values for all complications represent predicted probability and actual incidence as a percentage. Only length of stay is in days and calculated as a median. Medical complications with no c-statistic and low brier score reports did not occur in our cohort and were omitted from analysis.

**Table 5:** Complication predictions produced by the SpineSage^TM^ risk calculator.

Variable	Predicted Probability	Actual Incidence	Brier Score	C-Statistic
Any Complication (transfusions)	32	74.7 (62.1)	0.36	0.58
Serious Complications	7.2	1	0.02	0.5
Infection	12.3	2	0.02	0.6
Dural Tear	15.7	11.1	0.1	0.54

Note: Values for all complications represent predicted probability and actual incidence as a percentage.

## Data Availability

Patient data were compiled from patient charts; the data were then placed into tables for analysis. The data from this study are available upon reasonable request.
